# “But at home, with the midwife, you are a person”: experiences and impact of a new early postpartum home-based midwifery care model in the view of women in vulnerable family situations

**DOI:** 10.1186/s12913-023-09352-4

**Published:** 2023-04-19

**Authors:** Bettina Schwind, Elisabeth Zemp, Kristen Jafflin, Anna Späth, Monika Barth, Karen Maigetter, Sonja Merten, Elisabeth Kurth

**Affiliations:** 1 Swiss Tropical and Public Health Institute, Basel, Switzerland; 2 University of Basel, Basel, Switzerland; 3 Midwifery Network, Familystart beider Basel, Basel, Switzerland

**Keywords:** Early postpartum care, Midwifery, Home visits, Women’s experiences, Vulnerable family situations, Empowerment

## Abstract

**Background:**

Postpartum home-based midwifery care is covered by basic health insurance in Switzerland for all families with newborns but must be self-organized. To ensure access for all, Familystart, a network of self-employed midwives, launched a new care model in 2012 by ensuring the transition from hospital to home through cooperation with maternity hospitals in the Basel area. It has particularly improved the access to follow-up care for families in vulnerable situations needing support beyond basic services. In 2018, the SORGSAM (Support at the Start of Life) project was initiated by Familystart to enhance parental resources for better postpartum health outcomes for mothers and children through offering improved assistance to psychosocially and economically disadvantaged families. First, midwives have access to first-line telephone support to discuss challenging situations and required actions. Second, the SORGSAM hardship fund provides financial compensation to midwives for services not covered by basic health insurance. Third, women receive financial emergency support from the hardship fund.

**Aim:**

The aim was to explore how women living in vulnerable family situations experienced the new early postpartum home-based midwifery care model provided in the context of the SORGSAM project, and how they experienced its impact.

**Methods:**

Findings are reported from the qualitative part of the mixed-methods evaluation of the SORGSAM project. They are based on the results of seven semi-structured interviews with women who, due to a vulnerable family postpartum situation at home, received the SORGSAM support. Data were analyzed following thematic analysis.

**Results:**

Interviewed women experienced the early postpartum care at home, as “relieving and strengthening” in that midwives coordinated patient care that opened up access to appropriate community-based support services. The mothers expressed that they felt a reduction in stress, an increase in resilience, enhanced mothering skills, and greater parental resources. These were attributed to familiar and trusting relationships with their midwives where participants acknowledged deep gratitude.

**Conclusion:**

The findings show the high acceptance of the new early postpartum midwifery care model. These indicate how such a care model can improve the well-being of women in vulnerable family situations and may prevent early chronic stress in children.

**Supplementary Information:**

The online version contains supplementary material available at 10.1186/s12913-023-09352-4.

## Background

Based on evidence that lifelong health and human development are strongly influenced by experiences during the first years of life, early childhood interventions are increasingly the focus of research and policies [1–5]. Interventions during this period are found to be more effective and less costly than later efforts [3], and especially children in vulnerable family situations seem to profit from early interventions. Studies show in particular, that early chronic stress and its long-term consequences can be mitigated [6]. Evidence suggests that parents, caregivers, and families need support for providing responsive, nurturing care and protection for young children so that they may achieve their developmental potential [3, 7]. Programs designed to meet the needs of families in difficult circumstances lead to enhanced parental resources and thereby better outcomes for children [8–13].

Positive effects on developmental outcomes have been documented for three types of family support and strengthening: quality services, support, and skills building [3]. However, studies [14–20] have shown that especially psychosocially and economically disadvantaged families have limited access to postpartum care at home and use the available services less frequently. Midwives, due to their immediate access to vulnerable families, may therefore be key actors for early prevention, i.e. the early assessment and support of families with, or expecting an infant whose living situations are overstraining their capacities to cope [19, 21].

Only a few, mostly Scandinavian, studies have addressed the perspective of parents receiving postpartum home-based care by midwives, and research on the impact of such care on infants and their families is still scarce. A Swedish study on first-time parents’ experiences of home-based postpartum care after early hospital discharge showed that midwives took a supporting role and strengthened parents’ self-confidence [21]. In a Norwegian study on women’s experiences of home visits by midwives in the early postpartum period, three central themes, relational continuity, postpartum talk, and vulnerability emerged [22]. Specifically, relational continuity with a midwife appeared as a crucial part of care, as expressed by a cited quote “postpartum care provided by a named midwife”. The importance of relational continuity in care was supported by a study from the UK that linked care across pregnancy, birth, and new motherhood with improved health outcomes for women and babies in socially disadvantaged and diverse communities [23]. An Australian home visit program for vulnerable families in disadvantaged areas also improved clients’ parenting skills and well-being, increased participation in community networks, and access to support services [24]. In Sweden, parents in vulnerable situations who received extended home visits reported improved parenting skills and confidence in discussing problems with professionals [25], especially fathers with migration histories who benefited equally through home visiting programs [26]. Furthermore, a German study applying a longitudinal mixed-method design investigated the effects of family midwives in 734 vulnerable families in Sachsen-Anhalt [27]. Results showed an increase in mothers’ skills in three areas: childcare, self-help/organization of family life, and searching for and accepting external help.

Because the literature so far has largely focused on the context of extended home visiting programs and outcomes for families in vulnerable situations [25, 26] and was less concerned with experiences of families in vulnerable situations with very early home-based midwifery care and its impact, these issues were addressed in a Swiss study.

In Switzerland, early home-based postpartum care is mainly provided by independent midwives and family nurses [18]. Organizing postpartum care at home before birth is usually the responsibility of the pregnant woman and/or her relatives. Basic insurance covers 10–16 regular home visits by an independent midwife over 56 days. Little is known about the practices that go beyond standard care. Several local midwifery networks guarantee a seamless transition for all mothers and newborns from the hospital to the home setting [28, 29]. These networks coordinate a postpartum care pathway in collaboration with maternity hospitals, independent midwives, and other maternal and child health care providers [30–32]. They assure that all women who give birth in the collaborating hospitals receive standardized care in that a midwife comes to their homes after hospital discharge and ensures further care. A first evaluation study in Switzerland suggested a great value of organized, guaranteed postpartum outpatient care by a midwifery network, especially for socially disadvantaged families [16]. It appeared that the accessibility and reliability of the midwives were crucial to women. The midwife network not only eased the burden on families and reduced stress, and for many women, the midwife evolved into an important reference person and was recognized as a cultural mediator by women with migration history.

In the Basel area, a Familystart network model has been running since 2012. Due to the guaranteed access to postpartum home care, midwives regularly visit disadvantaged families who may have fewer resources to organize postpartum care themselves, yet need support beyond services covered through basic health insurance. Set up in late 2018, the project “SORGSAM – Support at the start of life” aimed to offer vulnerable families improved assistance in dealing with complex postpartum situations [33]. The SORGSAM project supports independent midwife care activities for families in situations of stress and risk in three ways: first, midwives have access to first-line telephone support (7 days a week) to discuss challenging situations and required actions with a midwife specialized in psychosocial care [33]; second, the SORGSAM hardship fund provides financial compensation to midwives for services not covered by basic health insurance, e.g. for their time and costs in emergencies or for coordinating inter-professional services; and third, women may receive financial emergency support from a hardship fund.

We specifically aimed to investigate how women in vulnerable family situations experienced early postpartum home-based care by independent midwives provided in the context of the SORGSAM project, and how they viewed the impact of obtained care.

## Methods

The study and this report were conducted following the Consolidated Criteria for Reporting Qualitative Research (COREQ) [34]. The research team of the SORGSAM evaluation consisted of an interdisciplinary team dealing with society and health care in Switzerland anchored at the Swiss Tropical and Public Health Institute, University of Basel [35]. Ethical approval by the Northwest Switzerland Ethics Committee was obtained before the start of the study (BASEC 2019–02030), and in an amendment during the COVID pandemic concerning the conduction of zoom interviews and directly contacting the families through the caring midwife.

### Research design

This article reports findings from the qualitative part of the mixed-methods evaluation of the SORGSAM project in the area of Basel, Switzerland [35]. It is based on the results of semi-structured interviews with women in a vulnerable family situation in the postpartum period who experienced home based support from a Familystart midwife. The midwife made use of the SORGSAM support, including coaching/counseling by a specialized midwife and financial support from the SORGSAM hardship fund.

Open-ended, narrative-generating interview questions were developed in consultation with the research team and included three thematic blocks: (1) perception of the postpartum situation, (2) perception of the midwife’s care, and (3) perception of the current situation. Towards the end of the interview, participants could talk freely about topics that were not previously addressed but were important to them. An additional document shows the interview guide in detail (additional file 1). For the purpose of publication the guide was translated from (Swiss) German into English.

### Sampling and recruitment

Criterion-based maximum variety sampling was used to select participants based on two available SORGSAM routine documentations, namely: (1) reports of the SORGSAM reimbursement from the hardship fund and, (2) reports of provision of first-line telephone support provided by a specialized midwife for colleagues encountering complex family situations. The criteria consisted of poverty, migrant history, single parent, health, and/or psychosocial stressors to maximize the diversity of complex family situations. Women were eligible to participate if they received care from a Familystart midwife who requested SORGSAM support in 2019. Individuals with severe mental illness, and/or receiving support from a midwife who were not Familystart members, and/or having language barriers were excluded. The selection based on the sampling criteria was documented, discussed, and validated by the team.

Eligible participants were approached by their midwives and informed about the study. When they were interested, the informed consent packages were sent via mail. They had sufficient time to read through the documents, clarify questions and consider whether they wanted to participate in the study. Written informed consent was obtained from all participants before the interview.

Among 55 eligible participants, nine agreed on a contact date for an interview, whereas 32 refused to participate or did not react; four persons showed insufficient language skills for interviewing, and 10 could not be reached. Two participants did not attend the agreed appointment due to the illness of a family member. As they were no longer reachable afterwards, this was considered as a withdrawal from participation. Once seven interviews were completed, recruitment had to be suspended due to the financial constraints of the project.

### Data collection

Between February and July 2020, a senior researcher with a background in health and social sciences conducted seven interviews in the area of Basel in the German language. Each participant was free to choose the place of the interview. The first interview was conducted in February 2020, face-to-face in a café. Due to the increasingly tense pandemic situation, all subsequent interviews were conducted virtually via Zoom. The virtual approach appeared to be convenient for participants, as they did not have to find childcare for their children. All interviews were audio-recorded and lasted approximately one hour. The semi-structured interview method with open-ended questions allowed delving into participants’ perspectives so that women could talk freely about their experiences. Observations on the research process including the pandemic situation were noted in a reflexive diary by the interviewer.

### Data analysis

After transcription of the interviews data were analyzed following Braun and Clarke’s thematic analysis [36, 37], a method designed for researching the views and experiences of research participants. The qualitative data analysis software package MAXQDA 2018 was used to support the analysis steps for coding following thematic analysis [36, 37]. These included familiarization with the data (step 1), assignment of preliminary codes (step 2), search for preliminary themes (step 3), review and definition of themes (steps 4 and 5) and provide a written record (step 6). At a stakeholder workshop, consisting of 12 participants, the themes identified through the analysis were presented, revisited, discussed, and validated. No themes were corrected or determined to be missing. Based on the results of the analysis, a thematic model was jointly developed by the research team that formed the basis of the results section.

## Results

### Participants

Of the seven participating women, five had a migration history (see Table 1). One participant was of Swiss nationality, and one woman was a cross-border commuter living in Germany. Their ages ranged from 23 to 44 years. Four women had a tertiary education and three had attended primary school. At the time of the interviews, only one woman was employed (100%), whereas two reported unemployment status and four reported not working and currently not seeking paid work. Four women were married; three were single or living alone. The partners of the four married women worked full-time.

**Table 1:** Demographic information on the women interviewed (n = 7)

	Age	Country of origin	Number of years in CH	Educational level	Employment	Civil Status	Employment level of partner
**1**	38	Spain	-	Tertiary	0%^1^	Married	100%
**2**	37	Columbia	1	Tertiary	0%^1^	Married	100%
**3**	36	Egypt	14	Primary	Unemployed	Single	
**4**	37	Sri Lanka	17	Primary	100%	Married	100%
**5**	44	Switzerland		Tertiary	0%^1^	Single	
**6**	23	Somalia	8	Primary	0%^1^	Single	
**7**	39	Germany	Cross-border commuter	Tertiary	Unemployed	Married	100%

^1^self-report of not being working and currently not seeking employment

### Research findings

Three themes emerged from the analysis of the interviews, each containing several sub-themes and respective codes:

1. complex postpartum situation,
2. comprehensive postpartum care, and
3. psychosocial relief and empowerment.

**Fig. 1:**
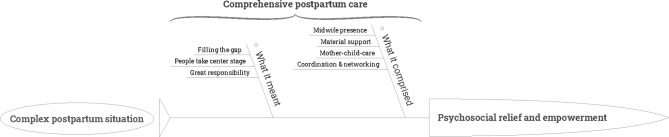
Thematic model

The themes were grouped into a thematic model as shown in Fig. 1, displaying midwife care and its perceived effects on women. They emerged from the accounts of the women, who found themselves in “complex postpartum situations” at home. Women described the “comprehensive postpartum care” by explaining “what it comprised” and “what it meant” to them. They indicated that the care received resulted in their “psychosocial relief and empowerment”. In the following, the three emergent themes are consecutively described:

### Theme 1: complex postpartum situation

**Fig. 2:**
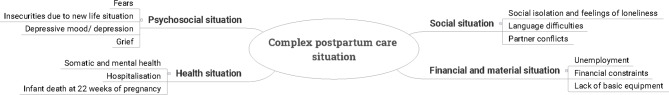
Theme “Complex postpartum situation”

Women described challenging situations at home. Four sub-themes became apparent, including the aspects: “health situation”, “social situation”, “psychosocial situation” and “financial and material situations” (see Fig. 2).

Specific to the present study, however, was that the postpartum situations were considered complex, due to the intersection of health challenges of mother and/or newborn, but also the precarious social and financial situation, partly with experience and/or fear of violence as the following example shows:

*“At the beginning, it wasn’t easy,…after the birth I had problems with my leg, I could not walk properly…and I am overweight…and I am here alone, without family. I had problems with my boyfriend at that time and with my boyfriend’s father, yes, and I have another child…and she was kidnapped when she was one year old, and the problems plus my fear that my new child would also be kidnapped, that made my life quite difficult.“* (Interview 3).

### Theme 2: “comprehensive postpartum care”

**Fig. 3:**
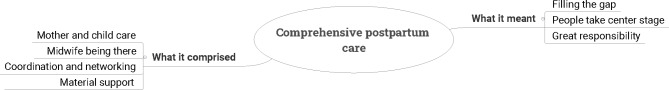
Theme “Comprehensive postpartum care”

Women reported how they experienced the care provided by midwives as shown in Fig. 3. Therewith, the sub-themes “what it comprised” and “what it meant” emerged from the data, each including different thematic aspects that added up to what was understood as “comprehensive postpartum care”.

#### Sub-theme “what it comprised”

The sub-theme “what it comprised” is composed of the thematic aspects of “mother-child-care”, “midwife presence”, “coordination and networking”, and “material support”. “Mother-child-care” included ordinary aspects of midwifery care in the postpartum situation, such as bathing the newborn, checking wound sutures, breastfeeding support, assuring weight gain of the newborn, and checking for emotional distress and/or anxiety/depression. To focus on the three further thematic aspects that go beyond ordinary midwifery care, mother-child care was not elaborated on in more detail.

“Midwife presence” emerged as a very central element in the women’s narratives. Women described the feeling that midwives were always there for them and their families, and even in case of emergencies:

*“She was always there when we needed her, always…” (Interview 1)*.

*“When I need help, when I write, she always calls.“ (Interview 4)*.

*“She really helped me and never minded, even if it was raining.“ (Interview 6)*.

Thus, the reachability and availability of midwives appeared as a crucial aspect to ensure emotional, parental and material support to promote feelings of security - especially in case of uncertainties at home. This was the case for example, when women were worrying about how to deal with the newborn, how to feed the baby, or if there were no nappies due to financial constraints, but also if they were fearing violence. This kind of accessibility and reliability to medical, social, and emotional support appeared especially important for women who felt challenged having to navigate through the health system and therefore they were able to receive the help at the time they needed it:

*“[Doctors] were often not available,…nobody came by…and I was…also not so well organized, but there was…a big gap…I somehow think that the midwifery care was a very personal, individual care.” (Interview 6)*.

The low threshold of accessibility and reliability, e.g. to text midwives and receive an answer via SMS, appeared as a supportive cornerstone. However, it was not only the perceived reachability and availability of the midwife that was important for the women, but also that the midwife’s presence at home was felt to be without any apparent time pressure:

*“I think it was important for us that she was simply there, that she always took her time, sometimes she was even there for two hours and didn’t look at the clock, … that she wasn’t in a hurry, but that she took her time and asked three more times, with, um, is it everything now, is there anything else?… Yes. That was good.“ (Interview 7)*.

The quotes suggest that the women perceived an unconditionality in the midwifery care received, presumably creating feelings of trust and being taken seriously. The women also reported that the midwife was not only there as a contact and care person for them and the newborn, but also for the whole family, as evidenced in the next quote of a woman who experienced stillbirth:

*“Yes, and I liked the fact that she looked after us as a family and not just after me as a woman, because there wasn’t much to control in terms of the baby…that the family was looked after, that the brother was looked after, asked how he was dealing with it, right? Things like that.“ (Interview 7)*.

Furthermore, one woman described a situation of domestic violence, in which her midwife provided her with emergency help:

*“Because once I was in a situation there, I couldn’t call the police or get help somehow, then I wrote to her and she got the police for me, you know?…I didn’t know what to do, I didn’t have a car, my baby was sick…so, or violence,…something happened to him,…I couldn’t go to the hospital right away, then she helped me, even though it wasn’t working time, or, so she came with her car, she helped me go to the hospital”. (Interview 6).*

Overall, the thematic aspect of “midwife presence” indicates that the interviewed women appreciated the midwife’s accessibility, availability, and continuity of care without time pressure. They described midwives as “carers” for their families and as trusted confidants - especially for themselves, and who were called in during emergencies such as in cases of domestic violence.

Women interviewed reported on the different forms of “coordination and networking” functions of midwives. They described midwives’ work as mediating, organizing, and coordinating services and institutions to improve complex domestic situations. Midwives were reported to have organized parent-child counseling, breastfeeding counseling, social services, and home care services for domestic help (e.g. the Red Cross). Women also indicated that midwives contacted various doctors, police, and cantonal offices. The women interviewed described this central interface function:

*“And, she gave me a lot of contacts…la Leche ligue, and now I’m a member and other mothers…that was very good.“ (Interview 1)*.

*“So yes, so I was sad, so not good, since the birth and yes, the midwife, has found such a person…a therapist, and I had gone there with (child).“ (Interview 2)*.

The range of this interface network function went from quick fixes to complex coordination activities as illustrated in the following example:

*“At the beginning, she just put away the toys…and then afterward she…asked at* Spitex [home care service for domestic help], *can someone cook there? And, do a bit of housework and a bit of cleaning. And they said, no, they just do the flat a bit. And then she …first asked in Canton X because the children were born there and then,…but I live in Canton Y…then she had asked if she could organize someone from the Red Cross if they would take over something. Then they said, no, have to ask Canton Y. She called Canton Y, and then they understood my situation, then Canton Y just took over and also organized it further.“ (Interview 4)*.

The midwifes’ networking activities included professional groups or organizations or institutions, and networking among mothers. Women with a migratory background who felt or were alone mentioned this positively and emphasized the important role of their home-country language in feeling understood:

*“Because the [other mothers] speak Spanish and it was…my midwife was the midwife of this mother and my midwife, and [she said] I know other Spanish mothers - do you want their phone number? And she asked for the other mother too, and we made contact and we are friends.“ (Interview 1)*.

Beyond midwife’s care and coordination support, women also described to have received material support, ranging from getting diapers to breast pumps to children’s clothes:

*“If I need some clothes or something bed and things like that…if I need that…all the organizing…for cot or something, you know…I didn’t buy much and she also tried to get [this] organized. That was also…great, how do you say, yeah because everybody thinks only about one health side, the other side…she helped on both sides, yeah.“ (Interview 4)*.

Women also received information and knowledge about where to obtain assistance in case of financial bottlenecks, e.g. where to get second-hand clothing and toys free.

#### Sub-theme “what it meant”

The sub-theme “what it meant” is composed of the thematic aspects of “filling the gap”, “people take center stage”, and “great responsibility”. The interviewed women described the overall postpartum care received not only as extensive, but also contrasted it with the care provided by medical doctors where they described midwives as “filling a gap” in the care system:

*“With the doctors a bit like that…so, ‘I do that, that’s my problem…They don’t see a collective problem…and he just looks at the child. I find the medicine a bit separate…The midwife! it’s in the middle…she’s worked with both of us so far, so that’s so her job, she’s doing great.“* (Interview 2).

Women also described as being seen and treated by the midwife as a person, which also included much of their emotional situation.

*“Because in hospital you are a patient with blood pressure, this and that and the values, but at home, with the midwife, you are a person with feelings and yes…that was a completely different approach.* (Interview 7).

*“I mean, it isn’t just the baby what the midwife works on…she helped me in other ways too.”* (Interview 6).

Women also emphasized the great responsibility that this entails for the midwife:

*“I felt that the midwife took more responsibility than she…had to.” (Interview 2)*.

The quotes highlighted that women understood the care they received as comprehensive postpartum care that occurred at the interface between somatic and psychosocial care, and as interconnecting between professions and institutions. This meant for women that a gap in postpartum care was being filled, which they described as a great responsibility for midwives.

### Theme 3: Psychosocial and emotional impact

**Fig. 4:**
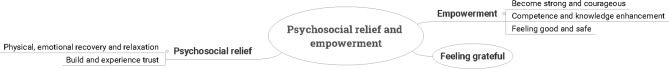
Theme “Psychosocial relief and empowerment”

The theme “psychosocial and emotional impact” developed from the data, covering the sub-themes “psychosocial relief”, “empowerment”, and “feeling grateful”, see Fig. 4.

#### Sub-theme “psychosocial relief”

What appeared as important for the interviewed women was that the received care was experienced as personal and emotional, and was labeled “human”. This aspect was particularly memorable for the interviewed women as they described the midwife’s care as supporting “physical, emotional recovery and relaxation” which helped them to relax:

*“Helped me to relax a bit, because I was always very stressed and so (groans), and I…was breastfeeding (child),…so and she helped me to relax a little bit like that, and to breathe…and to get a little bit, yeah, calm, so that was…that was good”. (Interview 2).*

*“It all sounds like a commercial now…she couldn’t have done it better. She was also great interpersonally…That really supported me insanely.“ (Interview 4)*.

The quotes indicated how women no longer felt alone due to the midwife’s presence and the interpersonal relationship, which supported women in dealing better with the new postpartum situation. They also stated how important it was for them to be able to “build and experience trust” with the midwife:

*“I could trust her…that was important for me, that I could trust her, I could also talk so openly with her.”* (Interview 6).

*“She was actually my contact person number one and I think that was actually almost the most important thing.”* (Interview 5).

The quotes underlined the importance of interpersonal closeness and trust so that women could open up and thereby feel relief and relaxation at the same time. They experienced a reduction of concerns and stress.

#### Sub-theme “empowerment”

From the different interview texts, it became manifest that the women not only felt relieved by the comprehensive midwifery care but that they also felt strengthened to survive the difficult life situations. The following quotes expressed how women felt to “become stronger and courageous”, also through “competence and knowledge enhancement” on where and how they could obtain help:

*“That made me a bit strong,…um, I don’t know, is there this expression in Switzerland or German, that you can stand on your feet? [Yes. That was like that for me. So I know where, where I can go for help if anything happens…yeah…And that made it a bit easier, the situation made that, became a bit easier. It’s not easy, but it has become easier.“ (Interview 3).*

*“During the birth or after the birth a little bit so, just, like nice, so, just, so, just, um, agreeing with the situation or so a little bit braver, we can help, we can organize something, not worrying or so a little bit, yeah, so just with the others, just helping would be nice (laughs)” (Interview 4).*

The midwifes’ presence provided security and confidence to better deal with and accept the current situation so that women described, “feeling good and safe”:

*“So yeah, now it’s, um, now it’s really like, I hope, I don’t know, but like…right now I’m somewhere good with my baby” (Interview 6).”*

Through these different forms of emotional and social self-empowerment, they described feeling “good and safe” again.

#### Sub-theme “feeling grateful”

Starting from the question about changes compared to the current situation, women made statements that indicated, in retrospect, a positive assessment and gratitude for the care and support they had received that still lasted at the time of the interview. This was exemplified in interview 6:

*“She just…helped a lot, a lot, and I’m very grateful that she kind of saved my life, twice.“ (Interview 5)*.

This gratitude was rooted in the comprehensive support, which was described as “coming from the heart”, as it was formulated in interview 5:

*“She helped me in many ways, you know…in clothes, in healthy, in emotional, she was … a person…she did so many things with me, helped…she did it from the heart.“ (Interview 5)*.

*“She has been like a god when she asked like that and organized the help like that, that’s, yeah, that’s a big help, you know? I won’t forget that.“ (Interview 3)*.

The quotes illustrated both the gratitude towards the midwife as well as her central role, possibly because the interviewed women have had little support in their vulnerable family situations.

## Discussion

Our study showed that women living in vulnerable family situations and who were cared for in the context of the SORGSAM project, evaluated the early postpartum home-based midwifery care as a relieving and strengthening experience. Midwives not only ensured the mother and child’s postpartum health but also coordinated further care and opened up access to appropriate community-based support services. Mothers described the received care as comprehensive, personal, and reliable, allowing them to better deal with complex family situations. They reported that it eased the burden of social isolation, made it easier to talk about challenges such as fears and violence, and led to de-escalation in situations of tension. They expressed how receiving care resulted in stress reduction, increased resilience, and empowerment and that it enhanced their mothering skills and parental resources.

Our study highlights that the benefits of early home-based midwifery care for women in precarious family situations are rooted in the close and trusting relationship with their midwife, resulting in deep gratitude for this experience. Participating women described their complex life situations and commented in detail on the supportive, comprehensive midwifery care they experienced. They reported that they felt strengthened by the continuous and easily accessible midwifery care during the uncertain phase of the early home transition, in terms of health, as well as social and emotional aspects.

”Midwife presence”, provided them with emotional and material equipment support, and supported them in accessing different community networks. This kind of supporting role by the midwife was described before, as in the study of Johansson in healthy families after early hospital discharge in Sweden [21], or in a recent Swedish study in families with low socioeconomic status, where the support of midwives was considered reassuring [25]. In our study, this aspect appeared to be very pronounced. Women expressed the impact of receiving midwifery care by memorable wordings such as “saved my life twice, somehow”, or “otherwise I would be dead”. Women included in the study used metaphors such as “angel” or even “God” to describe the midwives. Similar expressions by postpartum women have also been documented in a recent study in Zurich involving socially disadvantaged women [16]. These expressions may reflect, on the one hand, the hardships experienced in vulnerable situations and, on the other hand highlighted that support in such situations was perceived as particularly helpful. According to the Swiss study by Grylka-Baeschlin et al., midwives who provided postpartum care evolved into important support persons, and, among women with migration history, in that the midwives became cultural mediators [16]. Relational continuity has also been described as a crucial part of midwifery care in the review of Dahlberg [22], and it was linked to improved outcomes [23]. The high accessibility and reliability of the midwife appeared very central also in the other recent Swiss study [16, 38]. The building of trust probably occurred fully only if midwives could be immediately present in the homes of new mothers, recognize situations of psychosocial and emotional emergency, and act as first professional responders. As the mothers in our study reported, bridging to further help systems was a further, crucial part of care, and networking helped prevent social isolation. These findings are in line with those of a Swedish and an Australian study, that reported an increased knowledge of societal and local resources for families [26], increased access to support services, and improved participation in community networks [24].

Our findings exemplify that stress in the very first phase of life can be mitigated by early midwifery home care even in very difficult social situations. Mothers described impacts on themselves in terms of calming down, de-escalation of situations, strain relief, and stress reduction. Grylka-Baeschlin et al. found that home-based postpartum care eased the burden on families and reduced stress [16]. This should, in turn, positively impact mothering skills, the mother-infant relationships, and the further development of the children, as it has been shown, that a high level of parenting stress is associated with a poor dyadic co-regulation between mother and child [39]. “Midwife presence” seems to be helpful for what is called a “co-regulation in therapeutic processes” resulting in mitigating high-stress levels in the mother [40], which can be seen as a key factor for preventing/decreasing early chronic stress in the child.

A key finding of our study relates as to how early midwifery home care strengthened self-confidence and resilience, including knowledge and assurance on access to further support services. Women described that they felt to be seen and treated as “a person”, how they became ‘stronger and more courageous’. They furthermore reported increased competencies and knowledge of where to get help, and impressively described increased resilience. These findings *postpartum* are in line with several studies reporting that home-based early midwifery care had positive effects on parenting and self-confidence [21, 24–26, 38]. The mothers in our study also reported to have more confidence in that they now knew where and how to get the help they needed. It may thus be understood as a prerequisite for the promotion and prevention of mother and child health, including mental health aspects.

### Limitations and strengths

The positive picture given by postpartum women may be too optimistic due to a participation bias. Indeed, enormous efforts were needed for the recruitment of participants, and among the 55 eligible families, around half did not react to the study invitation or refused to participate. Ten women had no valid address, and four women had difficulty mastering the German language and this appeared to be too limited to participate in an interview. In particular, no woman could be recruited who was involved in a child welfare issue. Among the participants, six women had a migrant history, thus, migrant women were well represented.

Saturation is usually accepted as the criterion for the number of interviews conducted. Saturation is described to be reached - depending on the research questions and study population - at approximately 12 to 20 interviews, but the basic elements for a thematic ordination are reported to be already identified at six interviews [41]. Even though the sample was small by using a maximum variety sampling, we have succeeded in mapping the diversity and complexity of care for vulnerable postpartum family situations. The data collection was also quickly adapted to the pandemic measures and was carried out digitally. This actually was a simplified access for women in vulnerable family situations, as they were not burdened by the additionally needed childcare.

Furthermore, the information provided by the interviewed women was very congruent with the information in the midwives’ case documentation [35]. In these files, psychosocial problems were recorded in approximately 60% of cases, and midwives noted that they could contribute to a more stable situation, by lowering tension, exhaustion, and stress, and they notably documented a positive course in most of the cases. The thematic mapping was conducted by the research team and validated at the stakeholder workshop. Although there was no systematic assessment of the impact on children in our study, the findings suggested that SORGSAM care might influence favorably on children’s well-being as several of the interviewed mothers explicitly reported improved breastfeeding, and two out of the seven interviewed mothers mentioned a decrease in the crying of their children.

## Conclusion

In conclusion, our findings are supportive of a potential beneficial effect of postpartum midwifery care for the improvement of resilience and well-being of women in vulnerable family situations. Midwives appear to be important players in early childhood interventions with a comprehensive biopsychosocial approach breaching the interface of medical and psychosocial care. To allow midwives to make full use of their potential, it is necessary to install programs such as SORGSAM, which reimburse midwives for their coordinative services and give them access to a hardship fund enabling them to provide short-term financial support to families in acute need. Investing in midwifery services may be understood as a direct investment in earliest childhood interventions as described by Magistretti Meier [42] to prevent early chronic stress at early onset. The findings are suggesting that midwifery home care was a “door opener” for interprofessional coordinated early childhood support, strengthened parenting skills and self-confidence, and might alleviate early adverse childhood experiences, potentially reducing health care disparities and improving health equity. However, more studies are needed to quantitatively assess associations of midwifery home care with positive outcomes in mothers, and in particular, to assess and quantify its longer-term effects on the well-being of families, women, and particularly the development of children.

## Electronic supplementary material

Below is the link to the electronic supplementary material.

**Evaluation SORGSAM**: **Interview guide** with women experiencing vulnerable family situation postpartum at home

## Data Availability

The datasets produced and analyzed in this study are not publicly available due to the confidentiality of the information, especially in a small region like Basel, Switzerland, as this is the only way to ensure the non-identifiability of individuals. Upon reasonable request, the anonymized data are available from the authors.
