# Supplementary material for: “But at home, with the midwife, you are a person”: experiences and impact of a new early postpartum home-based midwifery care model in the view of women in vulnerable family situations
Source: BMC Health Serv Res. 2023 Apr 19;23:375. doi: 10.1186/s12913-023-09352-4 (PMC10114462; doi:10.1186/s12913-023-09352-4)
Supplement: Supplementary file 1 — Evaluation SORGSAM: Interview guide with women experiencing vulnerable family situation postpartum at home [file 12913_2023_9352_MOESM1_ESM.docx]

**Evaluation SORGSAM**

**Interview guide** with women experiencing vulnerable family situation postpartum at home

The interview guide was translated from (Swiss) German into English for this publication. Main questions are underlined. Following sub-questions, marked by an asterisk, are to be adapted to the specific situation.

1. **Warm-up and introduction, including ethical aspects**
2. **Main section**

- **Perception of the postpartum situation**

Can you describe to me what it was like for you when you came out of the hospital?

- Can you tell me what the situation was like?
- How did you feel?
- Thinking back, can you describe to me what was bothering you?
- So that I can imagine it, can you tell me more about it by giving me an example or telling me about a situation?
- How were you with your child?
- How was your child?
- How were you with your partner/husband/your parents?
- **Perception of care through midwife**

How did you experience the care you received from the midwife?

- Can you describe to me what the midwife did?
- What did the midwife do? How was this for you?
- Please tell me to what extent this was (important, difficult...) for you....
- So that I can imagine it, can you tell me more about it by using an example?
- **Perception of the situation today**

How are you today?

- Can you describe to me what the situation is like for you today?
- What is it like now?

1. **Cool-off and end**

Is there anything you would like to add to the conversation?

- Do you want to add something?
- Did you miss something?
- Did I not ask for something important to you?

Thank you for the interview.
